# Statistical process control to monitor use of a web‐based autoplanning tool

**DOI:** 10.1002/acm2.13803

**Published:** 2022-10-27

**Authors:** Hunter Mehrens, Raphael Douglas, Mary Gronberg, Kelly Nealon, Joy Zhang, Laurence Court

**Affiliations:** ^1^ Department of Radiation Physics The University of Texas MD Anderson Cancer Center Houston Texas USA; ^2^ The University of Texas MD Anderson Graduate School of Biomedical Science Houston Texas USA

**Keywords:** autocontouring, dose verification, statistical process control

## Abstract

**Purpose:**

To investigate the use of statistical process control (SPC) for quality assurance of an integrated web‐based autoplanning tool, Radiation Planning Assistant (RPA).

**Methods:**

Automatically generated plans were downloaded and imported into two treatment planning systems (TPSs), RayStation and Eclipse, in which they were recalculated using fixed monitor units. The recalculated plans were then uploaded back to the RPA, and the mean dose differences for each contour between the original RPA and the TPSs plans were calculated. SPC was used to characterize the RPA plans in terms of two comparisons: RayStation TPS versus RPA and Eclipse TPS versus RPA for three anatomical sites, and variations in the machine parameters dosimetric leaf gap (DLG) and multileaf collimator transmission factor (MLC‐TF) for two algorithms (Analytical Anisotropic Algorithm [AAA]) and Acuros in the Eclipse TPS. Overall, SPC was used to monitor the process of the RPA, while clinics would still perform their routine patient‐specific QA.

**Results:**

For RayStation, the average mean percent dose differences across all contours were 0.65% ± 1.05%, −2.09% ± 0.56%, and 0.28% ± 0.98% and average control limit ranges were 1.89% ± 1.32%, 2.16% ± 1.31%, and 2.65% ± 1.89% for the head and neck, cervix, and chest wall, respectively. In contrast, Eclipse's average mean percent dose differences across all contours were −0.62% ± 0.34%, 0.32% ± 0.23%, and −0.91% ± 0.98%, while average control limit ranges were 1.09% ± 0.77%, 3.69% ± 2.67%, 2.73% ± 1.86%, respectively. Averaging all contours and removing outliers, a 0% dose difference corresponded with a DLG value of 0.202 ± 0.019 cm and MLC‐TF value of 0.020 ± 0.001 for Acuros and a DLG value of 0.135 ± 0.031 cm and MLC‐TF value of 0.015 ± 0.001 for AAA.

**Conclusions:**

Differences in mean dose and control limits between RPA and two separately commissioned TPSs were determined. With varying control limits and means, SPC provides a flexible and useful process quality assurance tool for monitoring a complex automated system such as the RPA.

## 1 INTRODUCTION

Autocontouring and autoplanning tools are gaining prominence in radiotherapy (RT) research, in part because they may provide better access to RT globally.^1^, ^2^ The Radiation Planning Assistant (RPA) is an integrated web‐based autocontouring and autoplanning tool, currently under development, which provides plans calculated on standard linear accelerators (e.g., Varian 2100 using Golden Beam data).^3^ Users must then recalculate the plans in their own treatment planning systems (TPSs) for their own accelerators. Currently, the RPA provides autocontouring and autoplanning for complex plans, such as volumetric modulated arc therapy (VMAT) of the head and neck^4^, ^5^, ^6^ and postmastectomy RT to the tangential and supraclavicular fields with field‐in‐field segments^7^ (called “chest wall” hereafter), as well as simpler plans such as four‐field box RT of the uterine cervix^8^, ^9^, ^10^ and opposed lateral beam treatment of the whole brain.^11^

However, these techniques also require a rigorous quality assurance (QA) system to ensure safety is maintained and errors are identified within the process of the RPA before treatment delivery.^1^, ^2^ With such a complex system with diverse applications, an intuitive, flexible QA system is needed to monitor plans as they are calculated and exported from different TPSs. In addition, the purpose of this QA application needs to be focused on the process to ensure that communication of changes presented by both the RPA and the user's TPS do not introduce any additional errors. From a user's perspective, their standard QA procedure, which may include measurement‐based QA, such as gamma analysis for patient‐specific QA, has to still be performed outside of the RPA process to inform clinics of the acceptability of plans. Specifically, monitoring dose calculations in the user's TPS and comparing them with those calculated for standard linear accelerators by the RPA may identify unintentional errors or changes in the overall automated process or in the end user's systems. For example, changes in dosimetric settings in the end user's systems, such as the calibration of the planning system or changes to the multileaf collimator (MLC) parameters (e.g., dynamic leaf gap or transmission) may affect dose calculations. Other process changes, intentional or unintentional, that could result in dosimetric changes include changes to the CT‐electron density table used for dose calculations and changes to the threshold values used to determine the body contour.^12^ Because RPA plans are calculated twice—once by the RPA system and once by the end user's own TPS—QA systems for RPA should compare the two plans and identify differences or changes over time.

Statistical process control (SPC) has been previously used for dosimetric verification,^13^, ^14^, ^15^, ^16^, ^17^ patient‐specific QA,^18^, ^19^, ^20^ expansion of contour margins,^21^ verification of patient positioning,^22^ and evaluation of MLC and machine performance,^23^, ^24^, ^25^ in image‐guided RT^26^ and adaptive RT.^27^ The diverse use of SPC provides the flexibility to monitor the RPA's system. Understanding how SPC means and control limits are affected by differing TPSs and machine parameters may provide insight into how to monitor and assess errors in autocontouring and autoplanning systems. The purpose of this study was to determine the expected differences in means and control limits for different anatomical sites, contours, and machine parameters. Specifically, we used SPC to compare the mean percent dose differences and control limits of different TPS/algorithm combinations for three anatomical sites/treatment approaches (head and neck VMAT, cervix VMAT, and chest wall 3DRT) and machine parameters for head and neck VMAT plans (dosimetric leaf gap [DLG] and MLC transmission factor [MLC‐TF]). It is of note that the purpose of monitoring the mean percent dose differences for each contour is to identify gross changes throughout the RPA process.

## 2 METHODS

SPC has been used in several applications concerning RT and can help identify gross errors in the overall process. Specifically for our study, here are some examples of errors and their expected impact on the dose distributions that our QA might be able to observe: (a) changes in CT scanner, for example, poor description by the original CT number ‐ electron density tables; (b) changes in MLC parameters, for example, MLC‐TF or DLG, which could be changed unintentionally when local TPS is upgraded; and (c) changes in dose calculation algorithm used locally where beam characteristics are not well matched with the original dose calculation algorithm. Several task groups based on TPS commissioning and quality assurance, including TG‐53 and TG‐157, have shown that beam models can affect accuracy of dose calculations and supposedly, changes in these parameters either intentional or unintentional might produce further errors concerning the dose distribution.^28^, ^29^

The general workflow of the RPA begins with the upload of a CT scan and a service request form that contains pertinent information such as dose prescription and treatment approach. After the clinical user and radiation oncologist accept the prescription and approach, the RPA automatically performs contouring and planning. The RPA‐generated plan is then downloaded from the RPA website and imported into the user's TPS, in which the user recalculates the dose and completes any desired edits to the plan. Once finalized, the user‐calculated plan is uploaded back to the RPA for comparison with the original RPA plan. This workflow was designed to facilitate the deployment of the RPA to many centers without the need to specifically commission the RPA for local linear accelerators, thereby reducing overall running costs.^3^ However, the users still need their own TPS.

For this study, RPA plans, including the generated contours, were downloaded and imported into either RayStation v10B (RaySearch Laboratories) or Eclipse v13.6 (Varian) TPS (or both), mimicking an expected clinical workflow. The TPS was then used to recalculate the plans using fixed monitor units within contours provided by the RPA. Following the general workflow, the recalculated plans were uploaded back to the RPA, and the percent mean dose difference between the original RPA plan and the recalculated plan for each contour was calculated.

SPC was used to monitor the variations in percent mean dose difference for each contour. Upper control limits (UCL), lower control limits (LCL), and individual control charts were generated according to Equations (1) and (2)^30^:

(1) UCL=μ+3*σ

(2) LCL=μ−3*σ

where *μ* is the mean and *σ* is the standard deviation. Mean percent dose differences that fell outside of the calculated control limits were removed from the calculation of the mean and standard deviation.

The following RT plans were generated using the RPA (Varian Clinac): VMAT for head and neck cancers,^5^, ^6^ VMAT for cervical cancers,^9^, ^10^ and chest wall tangential and supraclavicular fields (with field‐in‐field segments).^7^ The previous references contain an in‐depth view and analysis of how these plans and contours are generated by the RPA.^5^, ^6^, ^7^, ^8^, ^9^, ^10^ Using the general workflow of the RPA previously mentioned, SPC was used to compare RPA through two methods: (i) anatomical and TPS/algorithmic differences, and (ii) machine parameter differences. It is important to note that the comparison by TPS/algorithm used separate systems, commissioned separately by different teams. This limits the true direct comparison of the TPS/algorithms; however, the flexibility of SPC, specifically its monitoring of trends over time and the process of the system, allows us to monitor changes based on their differences to identify potential errors or changes to the user's independent system. In addition, the anatomical comparison between metrics for the target and structures was based solely on anatomical regions and their corresponding structures that are supported by the RPA, no structural delineation comparison was performed.

### 2.1 Anatomical and TPS/algorithmic differences in control limits

To determine how control limits varied by the anatomical site and by differences in TPS and dose calculation algorithm, we generated 32 head and neck VMAT plans, 33 cervical four‐field box RT plans, and 51 chest wall plans with the RPA using an Analytical Anisotropic Algorithm (AAA) (Eclipse v.13.6). Specifically, the head and neck VMAT plans consisted of three arcs with energy of 6 MV, cervical four‐field box plans had an energy of 18 MV, and chest wall plans had 12 beams at energy of 6 MV and 18 MV with an additional two supraclavicular fields with energy of 18 MV. These plans were downloaded and imported into the TPSs. The dose calculation algorithm used by the RayStation v10B TPS was collapsed cone convolution (CCC), and the Eclipse v13.6 dose calculation algorithm was Acuros (dose to medium). The resulting plans were compared to the original RPA plans, and for each contour, a percent mean dose difference was calculated. Using Equations (1) and (2), control limits were calculated on the basis of these percent mean dose differences.

### 2.2 Machine parameter differences in control limits

To determine how control limits varied by machine parameter and dose calculation algorithm in the same TPS, we generated 30 head and neck VMAT plans using the RPA, downloaded them, and imported them into Eclipse v13.6. Dose calculation was performed on a different machine from that used to calculate the RPA plans and used either AAA or Acuros algorithm. In addition, DLG and MLC‐TF were varied for each plan. The choice of DLG and MLC‐TF was based on a study by Glenn et al.^31^ that found that for the Eclipse TPS, these parameters had the greatest impact on dose changes among treatment parameters. Using the 2.5th–97.5th percentiles of the values determined by Glenn et al., we selected five values for DLG (0.1, 0.155, 0.17, 0.19, 0.23 cm) and four values for MLC‐TF (0.0118, 0.0145, 0.0158, 0.0165).^31^, ^32^ The DLG and MLC‐TF values for RPA plans were 0.2 cm and 0.02, respectively. While the value of either DLG or MLC‐TF was varied, the other parameter value was held constant at that of the RPA plan. The resulting plans with variation in DLG and MLC‐TF values were compared to the original RPA plans (i.e., Eclipse‐AAA), and for each contour, a percent mean dose difference was calculated. Using Equations (1) and (2), control limits were calculated based on these percent mean dose differences. Linear regression was performed to characterize how the control limits and means changed with variations in DLG and MLC‐TF.

## 3 RESULTS

### 3.1 Anatomical and TPS/algorithmic differences in control limits

Table 1 provides the UCL, mean percent difference, and LCL for a selection of contours for each anatomical site. Specifically, head and neck and chest wall cases showed approximately 70% of contours being less than 2% in the mean differences between separately commissioned planning systems (RayStation‐CCC and Eclipse‐Acuros). All percent dose differences are relative to the automated plan.

**TABLE 1:** Percent mean dose differences from the RPA plan for selected contours from three anatomical sites

**Site/contour**	**RayStation‐CCC** mean (LCL, UCL)^a^	**Eclipse‐Acuros** mean (LCL, UCL)^a^
**Head and neck**		
Brain	−0.21 (−0.44, 0.01)	−0.24 (−0.42, −0.05)
CTV1	2.19 (1.04, 3.34)	−0.25 (−1.25, 0.75)
CTV2	1.26 (0.11, 2.40)	−0.22 (−0.72, 0.28)
CTV3	1.11 (−0.09, 2.31)	−0.36 (−0.82, 0.10)
Oral cavity	1.47 (0.24, 2.71)	−0.37 (−0.72, −0.02)
Left submandibular gland	1.71 (0.14, 3.27)	−0.94 (−1.59, −0.28)
Right submandibular gland	1.74 (0.45, 3.04)	−0.98 (−1.57, −0.39)
Larynx	2.28 (1.09, 3.47)	−0.97 (−1.57, −0.38)
Mandible	1.11 (0.20, 2.02)	−1.60 (−2.39, −0.81)
PTV1	2.29 (1.00, 3.59)	−0.48 (−1.83, 0.88)
PTV2	2.18 (−1.19, 5.55)	−0.15 (−0.68, 0.37)
PTV3	1.55 (−0.03, 3.14)	−0.32 (−0.74, 0.10)
Left parotid	0.49 (−0.61, 1.60)	−0.95 (−1.33, −0.57)
Right parotid	0.43 (−0.63, 1.49)	−0.93 (−1.20, −0.66)
**Cervix**		
Left femoral head	−1.78 (−2.78, −0.77)	0.21 (−0.92, 1.34)
Right femoral head	−1.79 (−2.81, −0.77)	0.19 (−0.93, 1.31)
L4	−2.21 (−4.87, 0.45)	0.49 (−4.43, 5.41)
L5	−2.59 (−3.43, −1.74)	0.48 (−1.31, 2.27)
**Chest wall**		
Clinical chest wall	1.51 (−1.65, 4.67)	−1.55 (−3.62, 0.52)
Clavicle	−0.86 (−2.20, 0.49)	−3.60 (−5.64, −1.56)
Heart	−0.17 (−0.68, 0.35)	−0.10 (−0.68, 0.49)
Humeral head	−0.34 (−1.18, 0.50)	−0.52 (−1.01, −0.03)
Left lung	−0.40 (−1.66, 0.86)	−0.36 (−1.97, 1.25)
Right lung	−0.34 (−1.69, 1.02)	−0.32 (−1.79, 1.15)
Ribcage	−0.17 (−1.05, 0.71)	−1.99 (3.59, −0.39)
Supraclavical	0.23 (−1.33, 1.78)	−1.21 (−2.69, 0.28)
Spinal canal	−0.17 (−0.51, 0.17)	−0.14 (−0.35, 0.07)
Sternum	0.61 (−0.56, 1.78)	−0.48 (−1.53, 0.57)

Abbreviations: CCC, collapsed cone convolution; CTV, clinical target volume; LCL, lower control limit; PTV, planning target volume; RPA, Radiation Planning Assistant; UCL, upper control limit.

^a^ All values are percentages.

**FIGURE 1:**
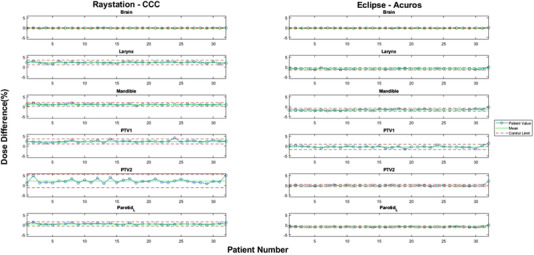
Selected individual control charts for head and neck contours. Left: percent dose difference between RPA and RayStation‐CCC. Right: percent dose difference between RPA and Eclipse‐Acuros. Blue circles: individual cases. Solid green lines: mean percent dose difference calculated by SPC for the indicated contours. Dotted red lines: upper and lower control limits calculated by SPC for the same contours. RPA, Radiation Planning Assistant; CCC, collapsed cone convolution; SPC, statistical process control; PTV, planning target volume

The individual control charts for head and neck contours showed that mean percent dose differences and control limits varied by structure and by the TPS/algorithm combination (Figure 1). In general, the differences for organs at risk (OARs) were smaller than those for targets. For RayStation‐CCC, the average range of the control limits (UCL−LCL) was 1.89% ± 1.32%, with an average mean percent dose difference across all contours of 0.65% ± 1.05%. In contrast, Eclipse‐Acuros’ average range was smaller, at 1.09% ± 0.77%, with an average mean percent dose difference across all contours of −0.62% ± 0.34%. Figure 1 shows example contours where this can be identified, especially in the primary target. Comparison of the two TPS/algorithm combinations showed that the average absolute difference in means was 1.31% ± 1.05% and the average absolute difference in range was 0.88% ± 1.12%. Overall, RayStation‐CCC produced a higher dose than did the RPA plan, whereas Eclipse‐Acuros produced a smaller dose than the RPA plan.

**FIGURE 2:**
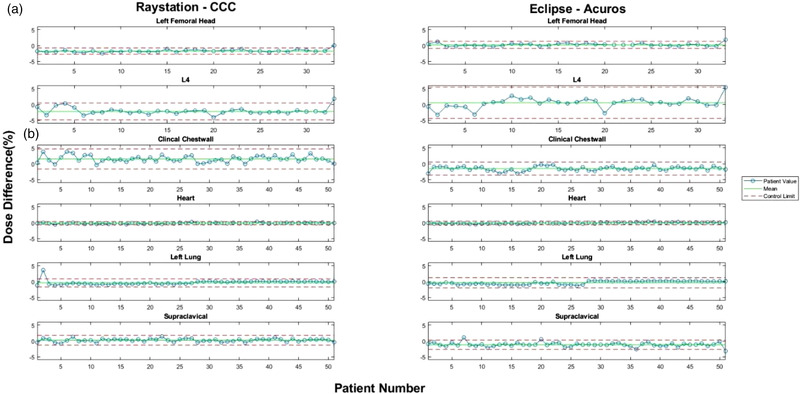
Selected individual control charts for (a) cervix and (b) chest wall contours. Left: percent dose difference between RPA and RayStation‐CCC. Right: percent dose difference between RPA and Eclipse‐Acuros. Blue circles: individual cases. Solid green lines: mean percent dose difference calculated by SPC for the indicated contours. Dotted red lines: upper and lower control limits calculated by SPC for the same contours. RPA, Radiation Planning Assistant; CCC, collapsed cone convolution; SPC, statistical process control

Unlike the head and neck contours, the mean percent dose differences for cervix contours showed larger and more consistent discrepancies between RPA and RayStation‐CCC as opposed to Eclipse‐Acuros plans (Figure 2a). The average mean percent dose difference for all cervix contours was −2.09% ± 0.56% for RayStation‐CCC, but only 0.32% ± 0.23% for Eclipse‐Acuros. Nonetheless, the average range of percent dose differences was larger for Eclipse‐Acuros, at 3.69% ± 2.67%, than for RayStation‐CCC, at 2.16% ± 1.31%. The average absolute difference in means across the cervix contours between RayStation‐CCC and Eclipse‐Acuros was 2.41% ± 0.77%, with an average absolute difference in range of 1.56% ± 1.49%. Unlike the head and neck plans, cervix plans with RayStation‐CCC produced a lower dose than the RPA plan, whereas Eclipse‐Acuros plans had a slightly higher dose than the RPA plan.

Finally, for chest wall plans with tangents and supraclavicular fields, we observed similar differences in means and control limits to those found in head and neck plans (Figure 2b). For RayStation‐CCC, the average range of the control limits was 2.65% ± 1.89%, with a small average mean percent dose difference across all contours of 0.28% ± 0.98%. In contrast, the average range of Eclipse‐Acuros was slightly larger, at 2.73% ± 1.86%, with a much larger average mean percent dose difference across all contours of −0.91% ± 0.98%. The average absolute difference in means across the chest wall contours between RayStation‐CCC and Eclipse‐Acuros was 1.25% ± 1.46%, with an average absolute difference in range of 0.83% ± 0.81%. However, in contrast to both head and neck and cervix plans, RayStation‐CCC and Eclipse‐Acuros plans both tended to produce a lower dose than the RPA plans. However, several contours, especially those generated by RayStation‐CCC, had a higher dose than that of the RPA, which made the overall average positive. As with the head and neck plans, this effect was associated more with targets than with OARs.

### 3.2 Machine parameter differences in control limits

**FIGURE 3:**
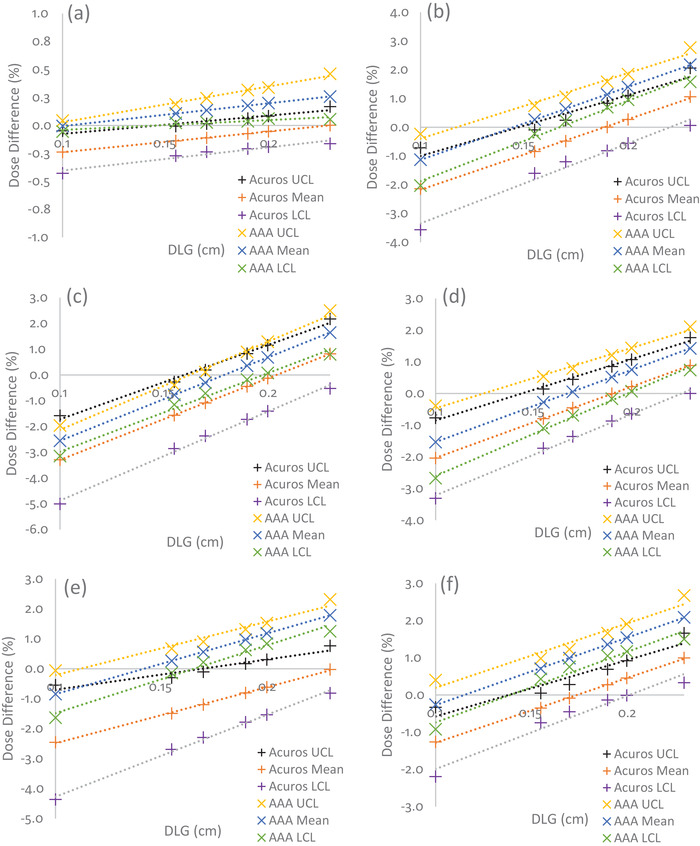
Percent dose differences with changes in DLG for head and neck plans calculated with different dose calculation algorithms: (a) brain, (b) larynx, (c) PTV1, (d) PTV2, (e) mandible, and (f) left parotid. The black, orange, and purple + symbols represent changes in Acuros’ UCL, mean, and LCL, respectively. Yellow, blue, and green x symbols represent changes in AAA's UCL, mean, and LCL, respectively. The dotted lines represent the linear fit. AAA had a narrower range between control limits than Acuros. For most contours, Acuros matched the RPA plan at an approximate DLG of 0.2 cm (removing outliers), while AAA matched the RPA plan at an approximate DLG of 0.14 cm. DLG, dosimetric leaf gap; PTV, planning target volume, UCL, upper control limit; LCL, lower control limit; AAA, Analytical Anisotropic Algorithm; RPA, Radiation Planning Assistant

**FIGURE 4:**
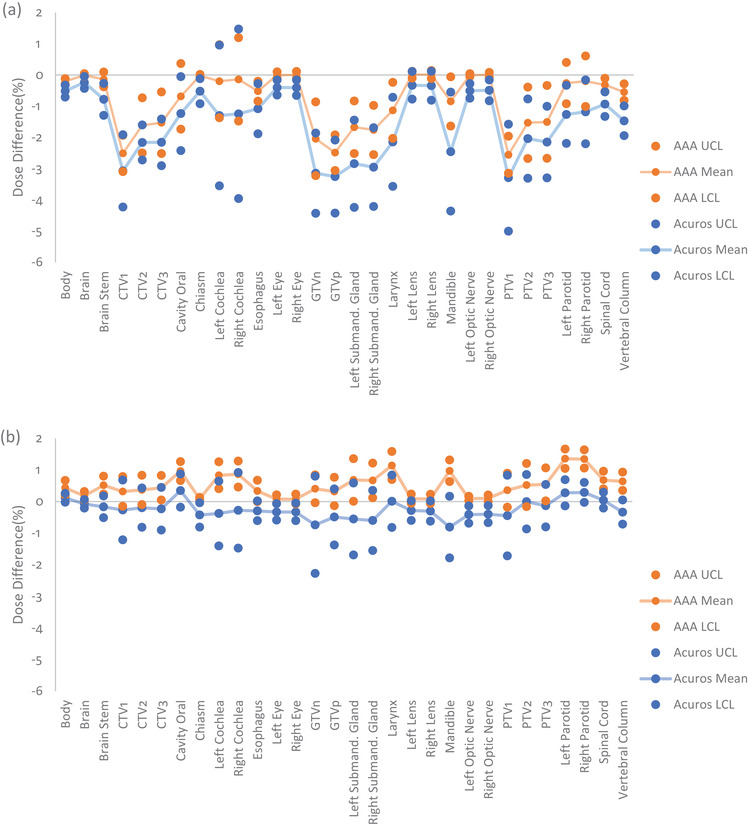
Mean percent dose differences, UCL, and LCL for head and neck contours calculated with AAA (orange) and Acuros (blue) at a DLG of (a) 0.1 cm, and (b) 0.19 cm. As DLG increased toward the value of the optimized DLG (0.2 cm) for the RPA machine, Acuros’ and AAA's means and control limits became more similar for all contours. UCL, upper control limit; LCL, lower control limit; AAA, Analytical Anisotropic Algorithm; DLG, dosimetric leaf gap; RPA, Radiation Planning Assistant; CTV, clinical target volume; GTVn, gross nodal tumor volume; GTVp, gross primary tumor volume; PTV, planning target volume

Figure 3 illustrates how the control limits and mean percent dose differences for the same six head and neck structures shown in Figure 1 changed along with DLG, and Table 2 provides the rate of change in the control limits and mean of the percent dose differences per 0.1 cm of DLG (and 0.01 of MLC‐TF) for Acuros and AAA. These rates of change values for DLG and MLC‐TF were chosen so as to discuss the largest variation observed based on what has been reported clinically for varying institutions.^31^ For Acuros, the control limits tended to shrink toward the mean as DLG increased. This feature is especially visible in Figure 3e for the mandible. However, for AAA, the rates of change for UCL, mean, and LCL were similar for five of the six structures, indicating that the control limits were relatively independent of DLG. However, there were exceptions, for example, the brain (Figure 3a), which showed a widening of control limits as DLG increased. On average for all contours, a 0% dose difference corresponded with a DLG value of 0.299 ± 0.180 cm for Acuros and 0.135 ± 0.031 cm for AAA. However, the Acuros value was greatly skewed by several outliers, including values greater than 0.5 cm for structures, such as the eye, lens, and optic nerve. After we removed these outliers, a 0% dose difference for Acuros corresponded with a DLG value of 0.202 ± 0.019 cm. The overall changes in control limits and means for AAA and Acuros for all contours for two DLG values (0.1 and 0.19 cm) can be found in Figure 4. A comparison of Figures 4a and 4b shows that targets’ means and control limits changed more dramatically as the DLG value increases. In contrast, the means and control limits of OAR structures tended to not change as dramatically.

**TABLE 2:** Rate of change of percent dose differences per 0.1 cm DLG and 0.01 MLC‐TF for selected contours of head and neck plans calculated by Acuros and AAA

	Rate of change of percent dose differences/0.1 cm of DLG						Rate of change of percent dose differences/0.01 of MLC‐TF					
	Acuros			**AAA**			**Acuros**			**AAA**		
	**UCL**	**Mean**	**LCL**	**UCL**	**Mean**	**LCL**	**UCL**	**Mean**	**LCL**	**UCL**	**Mean**	**LCL**
Brain	0.16%	0.19%	0.21%	0.32%	0.20%	0.09%	0.26%	0.42%	0.57%	0.44%	0.44%	0.44%
Larynx	2.12%	2.45%	2.78%	2.28%	2.54%	2.80%	2.46%	2.46%	2.37%	2.72%	2.49%	2.26%
Mandible	1.01%	1.87%	2.73%	1.78%	2.02%	2.26%	2.32%	2.28%	2.23%	2.47%	2.36%	2.26%
PTV1	2.88%	3.17%	3.45%	3.41%	3.23%	3.05%	1.87%	1.89%	1.91%	2.13%	1.93%	1.74%
PTV2	1.94%	2.25%	2.56%	1.90%	2.28%	2.65%	1.88%	1.85%	1.82%	1.95%	1.87%	1.83%
Left parotid	1.51%	1.74%	1.96%	1.72%	1.81%	1.89%	2.52%	2.54%	2.55%	2.59%	2.59%	2.58%

Abbreviations: AAA, Analytical Anisotropic Algorithm; DLG, dosimetric leaf gap; LCL, lower control limit; MLC‐TF, multileaf collimator transmission factor; PTV, planning target volume; UCL, upper control limit.

**FIGURE 5:**
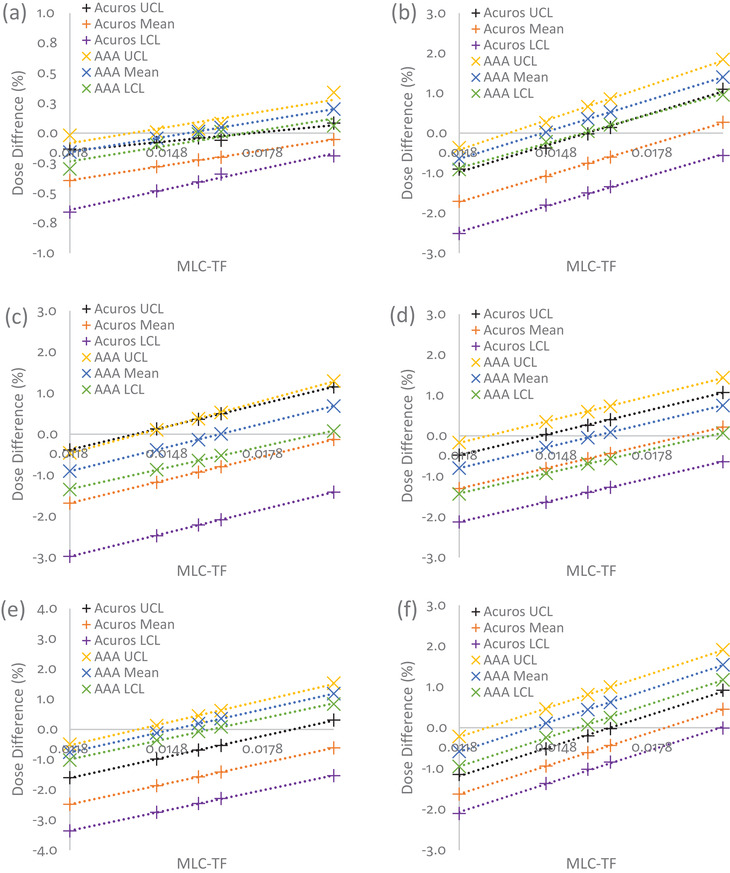
Percent dose differences with changes in MLC‐TF for head and neck plans calculated for different dose calculation algorithms: (a) brain, (b) larynx, (c) PTV1, (d) PTV2, (e) mandible, and (f) left parotid. The black, orange, and purple + symbols represent changes in Acuros’ UCL, mean, and LCL, respectively. Yellow, blue, and green x symbols represent changes in AAA's UCL, mean, and LCL, respectively. The dotted lines represent the linear fit. AAA had a narrower range between control limits than Acuros. For most contours, Acuros matched the RPA plan approximately at an MLC‐TF of 0.02, while AAA matched the RPA plan approximately at am MLC‐TF of 0.015. MLC‐TF, multileaf collimator transmission factor; PTV, planning target volume; UCL, upper control limit; LCL, lower control limit; AAA, Analytical Anisotropic Algorithm; RPA, Radiation Planning Assistant

**FIGURE 6:**
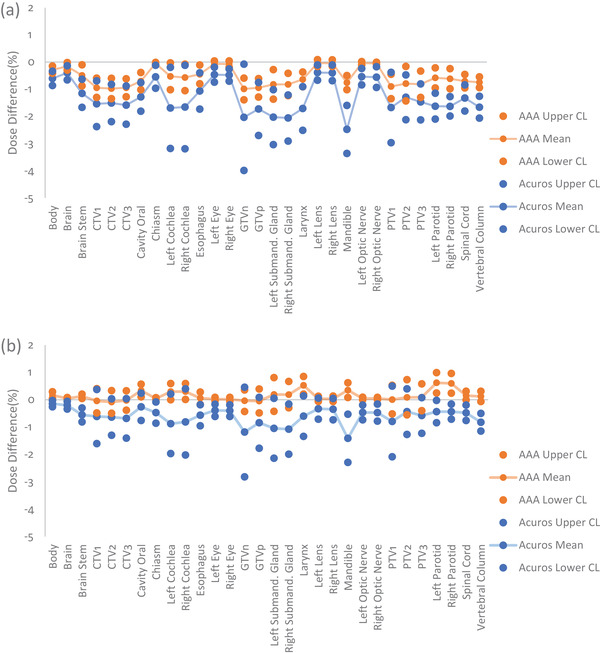
Mean percent dose differences, UCL, and LCL for head and neck contours calculated with AAA (orange) and Acuros (blue) with an MLC‐TF of (a) 0.0118, and (b) 0.0165. As MLC‐TF increased toward the value of the optimized MLC‐TF (0.020) for the RPA machine, Acuros’ and AAA's means and control limits became more similar for all contours; however, this effect was less pronounced than it was for DLG. UCL, upper control limit; LCL, lower control limit; AAA, Analytical Anisotropic Algorithm; MLC‐TF, multileaf collimator transmission factor; RPA, Radiation Planning Assistant; DLG, dosimetric leaf gap; CTV, clinical target volume; GTVn, gross nodal tumor volume; GTVp, gross primary tumor volume; PTV, planning target volume

For the same six head and neck structures, Figure 5 shows how changes in MLC‐TF affected mean dose differences. Unlike DLG, Acuros tended to show no consistent distinguishing pattern as MLC‐TF increased. This is especially apparent in a comparison of Figures 5a and 5b, which shows a decrease of control limits and no change in control limits as MLC‐TF increased, respectively. A similar pattern was observed for AAA. Only the control limits for planning target volume 1 (PTV1) showed a slight broadening with increasing MLC‐TF, while the control limits for the other contours remained steady, indicating, as with DLG, that the control limits were relatively independent of MLC‐TF. On average, for all contours, a 0% dose difference corresponded with an MLC‐TF value of 0.025 ± 0.010 for Acuros and 0.015 ± 0.001 for AAA. Again, we observed that this value for Acuros was skewed by several outliers for structures such as the eye, lens, and optic nerves, although the effect of these outliers was not as prominent as it was for DLG. After removal of the outliers, a 0% dose difference for Acuros corresponded to an MLC‐TF value of 0.020 ± 0.001. The overall changes in control limits and means for AAA and Acuros for all contours for two MLC‐TF values (0.0118 and 0.0165) are shown in Figure 6. However, in contrast with the pattern observed for DLG, we observed a consistent rise in the means for all contours as MLC‐TF increased, with the control limits at approximately the same range around the mean.

## 4 DISCUSSION

In general, several factors must be considered when using SPC to monitor a process. These can include, but are not limited to, the known potential systematic errors throughout a process and the effect of the process on the metric you are monitoring. Furthermore, the implementation of SPC is important considering which control charts might be used (e.g., individual control charts) and how to identify the number of points that are sufficient to categorize SPC's mean and standard deviation. However, for our specific case, SPC will be integrated into the RPA as a process QA monitoring system and the determination of these important considerations concerning SPC will be determined and adapted by the RPA staff. We aim to flag outlier situations to the user, rather than have them develop their own SPC. For further reference and a graphical representation of the RPA workflow, please see the several figures provided by Court et al.^3^

SPC allows for the monitoring of these systems over time and can alert the user or provider of these tools to systemic changes or errors that may affect plans. However, although a fixed threshold (e.g., 5%) may be sufficient to identify large errors, this approach may not be sufficient to capture the numerous potential errors that may arise during the complex process of autocontouring and autoplanning. Failure to identify small potential sources of error may lead to missed opportunities to identify larger, unintentional changes in how equipment and automated tools are being used. SPC can produce individualized results per contour, as shown in Table 1. Across different TPS/algorithm combinations and anatomical sites, the mean and range of the control limits varied from tenths of a percent to greater than 5%. This variability arose from several factors, including, but not limited to, TPS/algorithm dose calculation differences and calibration of machine parameters.

A phantom study by Alghamdi and Tajaldeen^33^ showed that CCC had better dose agreement in out‐of‐field points compared to AAA and Acuros, whereas it did not agree as well for in‐field points of varying clinical densities. Similarly, our data showed, across all examined anatomical sites, that the largest differences between RayStation‐CCC and Eclipse‐Acuros were in the doses to primary targets, followed by OARs located proximally to the primary target (e.g., oral cavity contour for head and neck, left and right femoral heads for cervix). The doses then began to equalize toward the distal OARs (e.g., brain contour for head and neck, heart contour for chest wall). Particularly for targets near the surface in head and neck cases, this variability in SPC control limits may be attributed in part to differences in dose calculations near the surface and to differing body contours between Eclipse and RayStation.^12^ These factors also may explain why the dose differences in chest wall cases and head and neck cases were similar among the various contours, but were not as pronounced in the cervix cases, in which the dose differences were more uniform across contours. In addition, this might also provide an explanation as to why we see the opposite effect of overall dose in Raystation‐CCC versus Eclipse‐Acuros for cervix plans compared to head and neck and chest wall plans.

The dependency of the plan on the choice of machine parameters can also play a role in determining acceptable control limits. We found that as DLG or MLC‐TF increased, both the mean and range of control limits changed for some contours. Specifically, we observed a smaller range between UCL and LCL with AAA than with Acuros. This is unsurprising because the RPA plan also used AAA, though calibrated to different machine parameters. However, this finding also demonstrates the need to set variable limits when monitoring a system like the RPA; different RT facilities use different TPS/algorithm combinations. Specifically, Martin‐Martin et al.^34^ found that AAA and Acuros can differ by up to 6.3% if not properly characterized for VMAT head and neck flattening filter‐free RT cases—well beyond a clinical threshold, though this does not necessarily reflect the quality of the plan.

As the RPA is multi‐institutional, it is imperative to design a flexible QA system that can adapt to differing setups and machine configurations. Toward this end, the mean and control limits depend on setup of the user's TPS; therefore, the SPC limits are institution specific. It is important to emphasize this fact of the SPC limits being institution specific, because the use of SPC is for the purpose of analyzing the process that invariably differs, sometimes largely, between institutions. Specifically, agreement of their calculations with the RPA will depend on how their TPS is commissioned, so SPC cannot be expected to identify commissioning errors, but can be expected to identify changes in the TPS.

Furthermore, we observed that not all machine parameters have the same impact across all contours. In particular, we observed that primary targets were the most affected when DLG was increased, in terms of both means and control limits. In contrast, increases in MLC‐TF tended to affect all contours similarly. In addition, AAA plans were less affected by changes in these machine parameters than were Acuros plans, for all contours. Nonetheless, Acuros and AAA became more similar as both DLG and MLC‐TF increased, specifically, when DLG was 0.2 cm and MLC‐TF 0.02, the values used by the RPA machine. However, we did observe outliers (e.g., eye, lens, and optic nerves) that skewed the value of MLC‐TF in which Acuros and RPA were equal. The increased sensitivity of dose changes to relatively small contours could explain why these contours were outliers and justify their removal from the calculation.

In this study, we investigated monitoring of mean dose differences. This choice was initially made so that anticipated differences between treatment planning systems (especially near the skin surface) would not overly affect our ability to monitor changes, particularly gross errors. Also, we did show that SPC can identify changes in process (e.g., in the user's planning system); however, one limitation of this work is that we did not investigate other metrics, such as several dose volume histogram metrics, maximum dose, or dose to the hottest 1 cm^3^. These other metrics might be more adept, when monitored through SPC, at identifying errors within the process and increasing sensitivity and specificity of our process QA. Further investigation is needed to identify the most appropriate parameter.

## 5 CONCLUSIONS

This study used SPC to determine the differences in mean dose and control limits between RPA and two separately commissioned TPSs. For head and neck and chest wall cases, most contours had only small mean differences under different TPS/algorithm combinations and machine parameters. Because it can account for the effects of varying control limits and means for different users and different planning approaches, SPC provides a flexible and useful QA tool for monitoring a complex autocontouring and autoplanning system such as the RPA.

## AUTHOR CONTRIBUTIONS

All the authors have contributed to the design, acquisition, analysis, or interpretation of the data for this work. In addition, all authors helped in the writing or revision of the manuscript, and approved the final draft for submission.

## CONFLICT OF INTEREST

The authors declare that there is no conflict of interest.
